# Interventions for reducing readmissions – are we barking up the right tree?

**DOI:** 10.1186/2045-4015-2-2

**Published:** 2013-01-23

**Authors:** Ran D Balicer, Efrat Shadmi, Avi Israeli

**Affiliations:** 1 Health Policy Planning department & Clalit Research Institute, Chief Physician Office, Clalit Health Services, Arlozorov 101, Tel Aviv, Israel; 2 Public Health Department, Faculty of Health Sciences, Ben-Gurion University, Beer-Sheva, Israel; 3 Faculty of Social Welfare and Health Sciences, University of Haifa, Haifa, 31905, Israel; 4 Ministry of Health, Ben Tabai 2, Jerusalem, 93591, Israel; 5 Hebrew University – Hadassah faculty of Medicine, Jerusalem, 91120, Israel

**Keywords:** Readmission, Quality of care, Health services research

## Abstract

Readmission reduction is at the focus of health care systems worldwide in efforts to improve efficiency across care settings. Yet, setting targets for readmission reduction is complicated due to inconsistencies in evidence pointing to effective organization-wide interventions and because of inverse incentives (such as maintaining high occupancy rates). Nonetheless, readmission reduction is one of the few quality measures that, if implemented properly, can serve as a catalyst for system integration. Appropriate mechanisms should be applied to hospitals as well as ambulatory settings to ensure that accountability is assigned to all stakeholders.

## Readmissions reduction as a re-emerging theme

Setting acceptable rates for readmission reduction and identification of effective interventions to achieve predetermined goals have been the focus of health care policy and practice for several decades. Recently, regulators in Europe, the United States, and Israel, have directed unprecedented incentives in an effort to tackle readmissions.

Several converging trends may explain why readmission reduction has become a focal point in health policy worldwide. Health systems are facing considerable strain associated with population aging and ensuing chronic disease at younger ages. These phenomena, coupled with the economic crisis, require health systems to increasingly pursue effectiveness and waste reduction in the management of population health. The promise of preventing a large proportion of the annual admissions is naturally quite compelling in these times.

In addition, the last decades have seen an increasing emphasis on quality measurement and quality improvement as key aspects of efficient health systems management worldwide. Variability in the rates of readmission [1] alludes to the potential for improvement in this care domain. Measurement of hospital readmission within 30 days of discharge is often used as a quality measure because it is relatively easy and inexpensive to measure, and may reflect poor quality of care during, or immediately after, discharge. Reducing the rates of readmissions, especially those that can be deemed as "avoidable", offers the prospect of improving care while simultaneously reducing health care costs. However, most of the current literature does not justify the use of rates of readmission as an indicator of the quality of hospital care [2,3].

Despite the lack of consensus in defining "avoidable" readmissions [4], it is generally agreed that about 10–20% of early (commonly measured as 30-day) readmissions are unjustified, suggesting that we should not overlook the importance of reducing unnecessary readmissions. The growing interest in readmission reduction, as reflected by recent international policy mainly in the U.S. and the U.K., is also evident in the proliferation of studies in this field, which has more than doubled in 2011–2012 compared to yearly rates of publication over the past decade.

## Readmission reduction – what really works?

The paper by Benbassat et al. [5] provides an important summary of decades of published assessments of the effectiveness of interventions for reducing readmissions in a multitude of health care settings (including hospitals and ambulatory care settings). With a wealth of published data, and a comprehensive compilation of systematic reviews markedly different from one another in methodology and setting, the paper [5] is an important tool for policymakers interested in the bottom line, i.e., What really works in reducing readmissions?

Yet, there are several issues and limitations that must be acknowledged when considering the translation of published evidence into actual health care policy and practice. First, given the breadth of literature in the area, a meta-review is an exceptionally valuable tool for summarizing findings and providing direction to evidence-based policy and practice. However, meta-reviews are contingent on studies previously summarized in meta-analyses and therefore are subject to a publication lag and are affected by the types of inclusion criteria employed in each of the summarized reviews. In the field of readmission interventions, recent studies have shed new light on the effectiveness of some integrative interventions. Moreover, replications of controlled trials in larger demonstration or prospective cohort studies provide invaluable evidence on the generalizability and large scale implacability of the suggested interventions. Recent Randomized Controlled Trials [6,7] that have been replicated in larger cohort studies [8,9] present the positive effects of an in-hospital discharge planning intervention, coupled with a post-discharge follow-up component.

Second, most studies of readmissions focus on single conditions as the reason for the index hospitalization and often limit the analysis to readmissions for clinically related reasons. Chronic heart failure (CHF), chronic obstructive pulmonary disease (COPD), and pneumonia are the three most commonly studied diseases associated with readmissions. This focus on a specific disease is clinically appealing, as the root cause related to the readmission is often sought. However, readmissions are rarely caused by factors solely related to the index admission [10] and multimorbidity is a major risk factor for readmission, calling for a broader, non-disease-specific assessment of the risks and solutions. A typical patient admitted with CHF could potentially have six additional serious chronic illnesses and complex socially related characteristics, which are just as likely causes of readmission.

Most importantly, too few studies to date have assessed the impact of multi-disciplinary integrated interventions extending from the hospital to the family physician practice and patient home. Most current literature focuses either on in-hospital [11] or ambulatory care [12,13] interventions. Yet, the breadth of risk factors identified in the literature, spanning health care setting characteristics [14], availability of health care resources [15], area level patterns of health care practice [16], and care trajectories [17,18] point to the need for developing and testing interventions in real world settings in various population groups. Moreover, very few interventions have been attempted in integrative care systems such as Clalit Health Services in Israel or Kaiser Permanente in the United States, where chances of successful transitional care may be a priori greater due to the integrated structure of primary, secondary, and tertiary care and supportive information technology (IT) systems in a single organization.

## Care transitions and readmissions – are we doing it right?

An especially vulnerable phase in the course of patient care is the transition between care settings [19], which necessitates integration of information exchange and clear delineation of continued care responsibility. It is well established that patients face significant challenges in understanding the numerous details involved in hospital discharge instructions, which are often provided over short periods of time [20,21]. Patients with multiple chronic conditions consume a majority of inpatient services and are especially vulnerable to breakdowns in care transitions, as their discharge instructions include managing multiple medications and treatments as part of their continuing care plans. With multimorbidity a consequential risk factor for emergency readmissions, it is evident that there are significant integration challenges associated with care transitions of these multi-morbid patients [22-24].

## Reducing readmissions – should we incentivize reduction via a quality measure?

With recent reforms enacted in the U.S. and other developed nations, one of the most widely used mechanisms to reduce readmissions is to establish targets for institutions (usually hospitals). Setting targets, deriving measurements, and introducing incentives through compensation or penalty are widely accepted approaches within the quality improvement context. Nonetheless, the usefulness of readmission rates as a quality indicator is much debated. Those in favor point to its simplicity of measurement and its comprehensibility and relevance for health care professionals as well as for policy and decision makers. Several additional factors point to the advantages in using readmission reduction as a quality indicator (or, when relevant, pay-for-performance target):

• Considerable differences are documented between health care organizations, even when adjusting for case-mix, providing evidence that at least some institutions exhibit relatively poor performance [1]. Some of these differences were shown to be closely associated with different incentives [14].

• Establishing targets and incentives to lower readmissions rates offers an additional balancing factor to the volume-driven incentives of fee-for-service care (as provided in most hospitals in western countries), where the financial incentive is to provide more care (i.e., admissions and overall stay). In the optimistic scenario, this will favor more value-based over volume-based health service provision.

• In the local Israeli setting, where hospital bed use is stretched to the limit with a 99% occupancy rate, much higher than the 76% OECD average (2010 data) [25], any reduction in admissions may reduce the burden from over-burdened wards and personnel.

• Attempts to reduce readmissions are likely to promote patient-centered care that crosses single institutions’ traditional boundaries. As health care systems still operate as separate silos, integration mechanisms, such as readmission assessment, may serve as an attractive vehicle by which incentives are "forced" to be aligned in efforts to achieve greater efficiency and seamless care.

Still, opponents of large-scale adoption of readmission as a quality indicator raise several concerns.

• Readmissions are not considered medical “never events”. As a considerable proportion of readmissions are unavoidable [4], overzealous attempts to block readmissions may become a hindrance to the delivery of proper medical care and to patient safety.

• There is no established definition of a “desirable” readmission rate, because many aspects of clinical and social case-mix have a profound impact on readmission rates, thereby making it very difficult to set goals for readmission rates. Improper adjustment may unfairly add financial burden to the institutions caring for the underprivileged and sicker populations and the appropriate approach to case-mix adjustments is still debated [26]. Additionally, instituting relative goals (reduction from year to year) tends to penalize the more effective systems that had lower initial adjusted rates.

• The trade-off of reduced readmissions could be in increased length of stay. Keeping the patient longer in the hospital may be attributable to attempts to provide comprehensive care within the hospital walls and to achieve better coordination with post-hospitalization services. Yet, increased length of stay may also be explained as a means for balancing reduced occupancy rates as a result of reduced readmissions.

## Policy and practice implications

Some of the abovementioned conceivable shortcomings of using the rate of readmission as a quality indicator can be tackled with proper planning and regulation, and allow most of the potential advantages to be gained while minimizing adverse effects. There are several examples from which lessons can be drawn to develop such policies. We discuss lessons from local experiences within the Israeli health care context as well as from the international literature.

Some characteristics of potentially effective policies may include:

• Start slow

Begin with a focused, smaller-size but high-risk subset of the entire re-admission group. In Israel, the choice was made by the Ministry of Health (MoH) to concentrate on internal medicine wards (which have a 50% higher adjusted risk for readmissions), and to limit interventions to patients with a length of stay of 2 nights or more. In the U.S., the focus was based on specific clinical diagnostic groups (such as CHF), a strategy that is less practical in Israel due to coding insufficiencies (timing and accuracy).

These subgroups may still be too large for intensive intervention; additional risk stratification and targeted intervention strategies may be key to successful ongoing intervention by healthcare suppliers.

• Set relative targets

The difficulty in creating fair risk adjustment mechanisms may result in inequitable target setting. The most sensible option that does not require adjustment would be a relative improvement goal [27]. As current evidence mostly points to success in narrowly defined populations [5], and large scale interventions rarely point to reduction of more than a few percentage points in the rate of readmissions [28], a goal of 2–4% per year would be reasonable. A 20% reduction in two years, as set by the Israel MoH, is likely to prove unachievable or non-sustainable for the larger health plans.

• Do not neglect the community side of the care transition

Incentives should include and even focus on the provider responsible for patient care after discharge, not only the admitting hospital [29]. In Israel, where there is a clear “home” for every patient for care provision once discharged (through mandatory coverage by one of four national health funds [30]), the decision was made to focus incentives and measurement on the health plans. In parallel, it is important to assure through regulation and incentives that the hospitals will collaborate with the health plans' efforts to improve transition care during and after the hospitalization, especially in view of the potential inherent disincentives.

• Monitor carefully for unanticipated adverse outcomes

Quality monitoring and especially implementation of incentives directed at readmission reduction should be designed in ways that account for the potential for increased length of stay, gaming through creative registration of non-accounted admitting wards, and other events that may follow from the law of unintended consequences.

## Conclusions

Readmission reduction is an important vehicle to improve efficiency across care settings, and is one of the few quality measures that, if implemented properly, can serve as a catalyst for system integration. Appropriate mechanisms should be applied to hospitals as well as ambulatory settings to ensure that accountability is assigned to all stakeholders. Importantly, competing interests should be resolved as the motivation to fill hospital beds is still, generally speaking, not offset by reduced readmission incentives [16].

Israel provides a unique setting of a strong primary care infrastructure coupled with a unique IT system – full EMR coverage of community healthcare clinics and an interoperable data-sharing system with key hospitals. The successful implementation of community care quality measurement and quality improvement infrastructure may serve as an excellent basis for implementing and assessing advanced interventions for readmission reduction [31]. In a state of acute bed shortage and a record-high bed occupancy in the internal wards, all incentives (including those of most hospitals) are aligned towards reducing preventable readmissions, as evident in the already relatively low readmission rates. Initial reports of effective community-hospital collaborations in tackling this complex problem are emerging.

Through sustained support provided by current incentives put forward by the Israeli government, we may indeed witness implementation of unique integrative models for tackling this global problem and, hopefully, unique success stories, emerging from the “start-up nation”.

## 

Commentary on the paper by Benbassat and Taragin: Effect of Generic Clinical Interventions on Hospital readmissions: a meta-review of published meta-analyses and narrative reviews.

## Competing interests

The authors declare that they have no competing interests.

## Authors’ information

Ran Balicer is an Associate Professor at the Department of Public Health at Ben-Gurion University of the Negev and Secretary of the Israel Public Health Physician Association. He serves as the director of the Clalit Research Institute and of the Health Policy Planning Department, Chief Physician’s Office, Clalit Health Services.

Efrat Shadmi is a Senior Lecturer at the Faculty of Social Welfare and Health Sciences at the University of Haifa, Israel. She is also a senior consultant to the Department of Health Policy Planning at Clalit Health Services and co-editor-in-chief of the *International Journal for Equity in Health*.

Avi Israeli is the Dr. Julien Rozan Professor of Family Medicine and Health Care at the Hebrew University – Hadassah Faculty of Medicine; Director of the Department of Health Policy, Health Care Management and Health Economics, Hebrew University – Hadassah Braun School of Public Health & Community Medicine; Chief Scientist of the Ministry of Health; and co-editor of the IJHPR.
